# Centring the voices of birth mothers who faced infant removal through the co-production of the MUMS@RISC artwork: an arts-based evaluation of trauma-informed research collaboration and participation

**DOI:** 10.1186/s40900-026-00966-6

**Published:** 2026-09-17

**Authors:** Kaat De Backer, Tonka Uzu, Brooklynn Reid, Erica Hayhoe, Angela Frazer-Wicks, Cassandra Lawrence, Pauline Bentley, Elsa Montgomery, Jane Sandall, Abigail Easter

**Affiliations:** 1 Department of Women and Children’s Health, School of Life Science and Medicine, King’s College London, London, UK; 2 Cambridge School of Art, Anglia Ruskin University, Cambridge, UK; 3 Expert by Experience, NA, England, UK; 4 Isaiah’s Wish CIC, London, UK; 5 Her Circle, North East England, UK; 6 Methodologies Division, Florence Nightingale Faculty of Nursing, Midwifery and Palliative Care, King’s College London, London, UK

**Keywords:** Co-producing artwork, Creative methods, Child protection, Patient and public involvement, Maternity service research

## Abstract

**Background:**

In the UK, newborn babies are a growing group of children entering the care system due to safeguarding concerns. Their mothers often experienced a life course of adversity and trauma, resulting in fractured trust in public services and health and social care professionals. Moreover, pregnant and postnatal women with involvement from child protection agencies are known to have increased risk of perinatal morbidity and mortality. To address the existing evidence gaps around perinatal healthcare and outcomes for this group of women, the MUMS@RISC study was designed, with cyclical involvement of an advisory group with women with lived experience.

**Methods:**

We adopted a trauma-informed approach to public and patient involvement and engagement, which was reflected in our study values, the study design, as well as in our dissemination strategy.

**Results:**

As part of the dissemination, a summary report with key research findings was co-produced, accompanied by artwork that was co-created by the advisory panel, the lead researcher and an artist. The step-by-step process of co-creation of the MUMS@RISC artwork allowed for gradual incorporation of feedback of the advisory panel, resulting in intricate layers of details and meaning in each of the illustrations.

**Conclusions:**

Our approach to trauma-informed research collaboration and participation demonstrates that involving people with traumatic lived experience in a robust, sensitive way can be done and strengthens the credibility, contextual accuracy and interpretative validity of research findings. Our evaluation of co-producing artwork as part of our dissemination strategy highlights the potentially healing and empowering nature of genuine and sensitive co-production, providing a rare opportunity to centre the voices of a marginalised group of mothers and reflect their stories within the wider research findings through art.

**Supplementary Information:**

The online version contains supplementary material available at https://doi.org/10.1186/s40900-026-00966-6.

## Abstract (plain language summary)

In many high-income countries around the world, child protection processes are in place to make sure babies grow up in a safe and nurturing environment. In the most concerning cases, a newborn baby may be separated from its mother shortly after being born. In England, the number of newborn babies being separated from their birth mother has grown in the last ten years. Many of these mothers had a difficult childhood themselves, and faced many difficult challenges in their life. The MUMS@RISC study is a research project that wanted to learn more about these mothers and the healthcare they received, so we could improve the support available to them. This paper explores the different ways in which the MUMS@RISC study involved women with this experience in the research process.The research team worked closely with an advisory panel of six women who had their baby removed after giving birth. Together with the researchers, the panel made decisions about the study’s design, language, and the values that were present throughout the study. This collaboration between the panel and the researchers was done in a sensitive way, to make sure the panel members felt safe, listened to, and the risk of causing more trauma was kept as low as possible. This paper explains how we did this and the different steps to co-create the MUMS@RISC artwork, which was used to share research findings with a wide, non-academic audience. Through a step-by-step process, we developed powerful images that reflected the most important research findings. The different steps are explained by two mothers who were on the advisory panel, the lead researcher, and a professional artist. Our approach shows that involving people with lived experience in a robust, sensitive way can make research findings more accurate and meaningful. The women involved in the advisory panel shared that the process was empowering and healing, and provided a rare opportunity to have their voices truly heard and their stories accurately told through art.

**Supplementary Information:**

The online version contains supplementary material available at https://doi.org/10.1186/s40900-026-00966-6.

## Background

Meaningful collaboration with public and patient contributors has been widely considered to improve the quality, rigour and impact of research studies [1–3]. Facilitating research collaboration with underserved and marginalised groups and communities may come with challenges, due to previous experiences of exploitation, colonialism, oppression, discrimination and trauma [2]. One such marginalised group is mothers who had a baby removed by family courts shortly after birth due to safeguarding concerns, a process also known in the UK as *care proceedings*. In the last decade, numbers of babies entering state care shortly after birth have been rapidly increasing in the UK, as in many other countries. A recent study showed that in 2022/2023 in England 5,354 infants (under one year of age) entered care proceedings, and 344 in Wales. While this was a slight decrease from 2019/2020, the proportion of babies less than two weeks old had increased in England to 55% among all infants in care proceedings, rising from 41% in 2015/2016 and 52% in 2019/2020 [4].

Evidence has shown that many women facing separation from their newborn baby have experienced a childhood marked by neglect, abuse and trauma, and present with a range of ongoing social risk factors [5–7]. The trust placed in institutions and public services, also known as *epistemic trust*, is oftentimes eroded due to previous experiences with child protection agencies, for instance in the context of previous child removal [8]. The loss of epistemic trust is experienced as *professional betrayal*, as these mothers perceive their trust in health and social care professionals during antenatal healthcare encounters and child protection assessments to be violated when infant removal occurs [9, 10].

On a societal level, mothers who have a child removed are often deemed unfit to parent, and therefore viewed as deeply flawed [11, 12]. Stigma and shame are prominently documented themes in qualitative studies with birth mothers who experienced infant removal [13]. Separation at birth has been described as a deeply distressing, intrusive and emotionally impactful event, leaving women to feel ashamed, rejected and stigmatized [12, 14]. Perceived judgement by health and social care professionals poses barriers to accessing, engaging with and receiving appropriate healthcare [13, 15–17] and can further exacerbate poor health. International studies on maternal morbidity and mortality have consistently identified this group of mothers as a particularly vulnerable group, with increased risk of suicide and other avoidable causes of death [18–20]. By consequence, more research is needed into this group of perinatal women, to improve maternal and neonatal outcomes and reduce existing health disparities.

In recent years, a range of clinical and policy documents have urged the need for trauma-informed approaches in clinical care for this group of mothers [21–23]. The commonly used definition of trauma-informed care was first developed by the US Substance Abuse and Mental Health Services Administrations (SAMHSA). Their 2012 guidance argued for embedding a trauma-informed approach in behaviour health services (in particular for those using substances) incorporating the four key principles of (1) *realising* the prevalence of trauma; (2) *recognising* how trauma affects individuals; (3) actively *resisting* re-traumatisation through carefully considered processes and settings; (4) *responding* by putting this knowledge into practice [24]. By doing so, the guidance encouraged healthcare providers to move away from stigma and judgement and towards a genuine, compassionate understanding of some challenging behaviours and coping strategies among drug and alcohol service users. Since the introduction of this definition, a range of further guidelines have been developed to ensure these principles are embedded in everyday clinical practice, often applied to specific settings or services, including in maternity and perinatal health settings [22, 25–29]. In line with recent guidance regarding the clinical care of these mothers [21–23], trauma-informed approaches to research collaboration as well as research participation are crucial when conducting research in this field [2, 30]. Trauma-informed *research collaboration* acknowledges that genuine, inclusive involvement of those with lived experience will improve the quality, transparency, relevance and accountability of research. In addition, it supports the safety of both researchers and the public members contributing to the research [3]. Similarly, trauma-informed *research participation* upholds trauma-informed principles in decisions about how to conduct research, how to engage with research participants, and how researchers commit to self-reflection and self-care during the research process [30]. However, a recent systematic review into healthcare experiences of pregnant and postnatal women with child protection agency involvement highlighted the paucity of research studies that had embedded rigorous trauma-informed approaches towards research collaboration and participation throughout the research cycle [9]. Nevertheless, trauma-informed research collaboration and participation are crucial to ensure (a) those who experienced infant removal feel safe and supported to shape and participate in research; (b) to avoid re-traumatisation and exacerbation of feelings of professional betrayal; (c) to rebuild epistemic trust.

The MUMS@RISC study is a doctoral research study, funded by the National Institute for Health and Social Care (NIHR302565), aimed at understanding the characteristics, outcomes and experiences of healthcare among women with child protection agency involvement during pregnancy and the postnatal period. Research findings from the different components of the convergent mixed-methods study have been published elsewhere [9, 15, 31–35]. This paper aims to describe the trauma-informed methods of PPIE that underpinned the MUMS@RISC study, with the creation of artwork as a worked example of trauma-informed co-design.

## Methods

The MUMS@RISC study adopted a transformative research paradigm, described by Mertens (2007, 2010, 2025) as research that is focused on the need for social justice and the pursuit of human rights, especially for communities at the margins of society [36–38]. By doing so, research can contribute “to address issues of discrimination and oppression and to support an increase in social, economic and environmental justice” (p.2) [38]. To achieve this goal, a transformative research approach embeds culturally competent, mixed methods strategies, with a strong participatory component, i.e. involving public contributors with lived experience as well as participants at all stages of the research and engaging in cyclical reviews of results [39, 40]. Mertens (2025) argues that it falls within the researcher’s remit to develop “processes that address inequities and provide reciprocity to the community members in terms of increased capacity to be a part of the research decision making” (p.2) [38]. Aligned with this, are the transformative epistemological and ontological assumptions, which expect the researcher to critically interrogate various different beliefs, especially those that emanate from oppressive positions of power and sustain oppression [40]. Knowledge production is not neutral and is influenced by human interest and position and reflects power and social relationships within society [39].

These assumptions profoundly influenced the entire study design, by involving lived experience voices cyclically throughout the research cycle, as well as in our dissemination strategy of research findings with a wider, non-specialist audience. Traditionally, clinically applied research studies predominantly share study findings through conference presentations and publications, aimed at an academic and clinical audience. However, such a dissemination approach would go against the epistemological principles of the MUMS@RISC study, as it reinforces existing power imbalances about knowledge production. Findings about women who have their baby removed at birth, had to be available to these women and the organisations that support them, including health and social care professionals, in ways that are accessible, understandable and meaningful. In the next few paragraphs, the different steps to achieve meaningful and trauma-informed research collaboration will be described. We subsequently will explore the iterative co-production of the MUMS@RISC artwork, representing the viewpoints of researcher, lived experience voices and illustrator.

### Composition of the MUMS@RISC advisory team

At the heart of the MUMS@RISC study, and in line with Mertens’ transformative paradigm was the MUMS@RISC advisory panel, consisting of six women with lived experience of infant removal. Women were recruited to the panel with the support of third sector organisations that support women who face infant removal. An information leaflet (see Supplementary materials S1) was provided to each of the third sector partners, with a request to share this with women on their caseloads that might be interested in joining the panel. The designated contact person in each of the third sector organisations was also asked to consider women’s wellbeing and availability when approaching them. For example, if women had recently experienced a removal, or had heard about a final adoption date of their child, it was deemed inappropriate to seek their involvement for this research study, due to its potential to be triggering. It was at the discretion of the contact person and the practitioners in each third sector organisation to assess who they felt would be potentially interested and able to be involved. Once initial contact was made with interested women, an individual meeting was set up with the researcher, to explain the aim of the study, its duration (three years), the role of the advisory panel, the frequency and length of panel meetings (6–8 weekly, 2 h, online) and the different options for reimbursement of women’s time, childcare expenses, mobile phone data and any other expenses they might incur as a result of their involvement. A total of six women decided to join the panel and remained involved throughout the entire study.

Panel members had diverse ethnic and cultural backgrounds, with one third of the members being from Black or Asian communities, lived in different parts of the country, and had diverse experiences of infant removal, whereby some of the mothers had their child returned to them, others had their child adopted, or remained separated from their child through a kinship care arrangement. The advisory panel, facilitated by the lead researcher of the MUMS@RISC study, met regularly throughout the course of the study, with an average interval of two months in between online meetings. Panel members were reimbursed for their time in accordance with NIHR INVOLVE rates (via bank transfer or shopping vouchers of their choice), with additional expenses for childcare costs and mobile data (if applicable).

### Co-producing the MUMS@RISC charter for research engagement

To create a safe and reflective space for the entire study project, we dedicated the first meeting of the MUMS@RISC advisory panel to explore the panel members’ views on how to conduct research in this context. It was vital to hear from the women what they envisioned the research could and should look like and what values and principles that we, as a collective of researcher and public contributors, aimed to uphold. The discussions were formatted in the MUMS@RISC Charter for research engagement, capturing the ethical principles that underpin the entire project, and inspired by the Survivors’ Trust research charter [41].

During the second meeting, the draft charter was presented to the group for validity checking and further input. By taking the time to have these initial discussions about the ethical underpinnings of the research, rather than diving straight into discussing research design, the panel started to get to know each other, in a safe and paced way. It also formed a crucial step for the women to get to trust the lead researcher, who had no lived experience of infant removal. Seeing how the draft charter reflected those early discussions, while using the women’s own words, was for some a novel experience, contrasting their previous experiences of seeing their words changed, redacted, or manipulated, often in the context of child protection assessments and family court hearings.

The final and approved MUMS@RISC charter for research engagement is available in the supplementary materials S2, publicly available online, and has been shared at several events with other researchers and practitioners.

### Ensuring a safe, empowering space

During the first MUMS@RISC meeting, a group agreement was also discussed to ensure meetings were a safe, confidential and respectful space at all times. The panel agreed on some routine checks and processes to ensure panel meetings were safe and trauma-informed, as presented in Table 1.

**Table 1:** Group agreement

1) A reminder is sent a few days before the meeting, to create headspace to attend.
2) At the start of each meeting, all participants are invited to self-score on a scale of 0 to 10 on how they are feeling that day, with 10 being perfectly happy, and 0 being the most worrying.
3) At the end of the meeting, a second check-in is provided, where everyone is invited to give another indication of how they are feeling. This helped the lead researcher to understand the impact of the meeting on each of the participants, and helped to identify those that would benefit from a one-to-one debrief after the meeting. When a panel member reported a decrease in wellbeing self-score, the lead researcher would make contact with them via telephone or video-call immediately after the meeting to offer the opportunity for a one-to-one debrief. If needed, further signposting or escalation would be discussed with the panel member, until the researcher and panel member were both satisfied that it was safe to conclude the phone call or video-call. As relationships of trust developed throughout the project, panel members also remained in contact with the researcher more frequently through text message, which was helpful to conduct continuous (informal) check-ins in between meetings. At no point was further escalation required.
4) Meetings are not recorded but brief notes are taken during the conversations and shared with participants afterwards for sense-checking.
5) An agenda for the meeting is shared in advance and deliberately kept very brief, to allow conversations to flow freely.

### Designing the MUMS@RISC study

Feedback from the panel at each meeting was documented in a research collaboration decision log, to reflect any changes that were made to the study design, language, recruitment strategy, sense-checking findings, and dissemination strategies as a direct consequence of the panel’s feedback.

Table 2 gives an overview of some of the crucial input of the panel in each of the research studies, based on notes in the research collaboration decision log.

**Table 2:** Overview of the advisory panel’s input in the MUMS@RISC study

Research study	Research design	Language and recruitment	Interpretation and dissemination of findings
Systematic Review and Critical Interpretative Synthesis [9]	The panel insisted that a synthesis ideally was going to be more than a summary of different study findings. This feedback supported the decision to use Critical Interpretative Synthesis, rather than a Thematic Synthesis.	The panel reviewed the key findings and helped to name the corresponding synthesising theoretical constructs.	The visual display of review findings was discussed and co-designed with the panel. Any further feedback was incorporated in the final version.
eLIXIR-Born in South London prospective cohort study [33]	The panel provided input on how best to scope for certain risk factors with the variables available in the dataset	Not applicable in view of retrospective cohort	Findings were discussed with the panel. The panel themselves found findings about the impact of previous contact with children’s social care particularly striking, especially for women who were care leavers.
MBRRACE-UK cohort study and confidential enquiry [15, 32]	A panel member served as the lived experience voice on the wider steering panel. The data extraction template was co-designed with the panel, including prompts to ensure assessors were taken a consistent approach to data extraction.	The decision to include all causes of death for early maternal death sampling was based on the lived experience of multiple panel members, whose medical symptoms were overlooked during pregnancy and early motherhood in view of their increased social risk.	Preliminary findings were discussed and sense-checked with the MUMS@RISC advisory panel. Additional lived experience input was sought through the Lived Experience Team at Birth Companions and the HOPE mothers. The visual representation of findings was discussed and co-designed with the panel.
Narrative study [34]	The trauma-informed study design was informed by lived experience. The panel provided strong guidance to approach interviews as a whole, and to avoid a reductive approach to data analysis and potential fragmentation of interview data. This discussion led to the adoption of a narrative approach. The panel insisted on returning the transcript by default, in paper copy, to maximise women’s agency during their research participation.	The panel co-designed the safety protocol, recruitment strategy, and recruitment materials. The safety protocol detailed the various situations that may give rise to concern about participant wellbeing, and the appropriate steps to mitigate or escalate and formed an integral part of the ethical approval application. They guided the wording in the recruitment flyer (for example: “We want to hear your story.”) and in the Participant Information Sheet (for example using the word ‘conversation’, rather than ‘interview’).	The panel insisted on returning core stories to individual participants. Findings were discussed with the lived experience panel and sense-checked by participants. Participants chose their own pseudonyms and signed-off manuscripts prior to submission.

### A worked example of trauma-informed research collaboration: co-producing the MUMS@RISC summary report and artwork

In line with the feminist epistemological underpinnings of this research, it was essential that the knowledge derived from this research was widely available, including to a non-academic audience, in order to increase its impact. For this reason, a summary report, aimed at policy makers, practitioners in health and social care, commissioners, third sector organisations and families, was co-produced with the MUMS@RISC advisory panel [18]. As the study had a mixed-methods approach, the panel felt strongly that presenting overarching key messages across the different studies would be more impactful than a list of key study findings for each of the different studies. To ensure the report would be visually inviting and impactful, artwork was co-produced in collaboration with the advisory panel and a professional artist to enhance the key messages.

Through the regular meetings with the advisory panel and the lead researcher, consensus was reached on seven key messages to structure the summary report, bringing together research findings from the different mixed methods studies. Each of the seven messages would be accompanied by a co-produced image. The co-production of one of these images will be further explored in depth. The message “*The past does not stay in the past*” brought together research findings that demonstrated how childhood adversity continues to impact on women’s health outcomes and experiences of healthcare during pregnancy and early motherhood. Iterations of two further illustrations are available in supplementary materials S4.

Co-producing the artwork was an iterative step-by-step process, requiring the input of three main contributors:

- The lead researcher, to ensure that the artwork was accurately reflecting the study findings.
- The MUMS@RISC advisory panel, to ensure the artwork was rooted in lived experience, sensitive, and trauma-informed. The views of the panel, consisting of six women, have been presented by two panel members (Panel Member A and Panel Member B).
- The artist, to incorporate the feedback from the latter two into powerful artwork.

In the next few paragraphs, each of the contributors will describe their own reflections on the co-production process of the MUMS@RISC artwork. This was achieved through collating the written reflections of each of the contributors (researcher, artist and two panel members), as well as through sense-checking by three other panel members of the MUMS@RISC advisory panel.

### Phase 1 - Brainstorm and preparation

**Researcher**

*During an initial explorative meeting with the illustrator*,* I explained the aims and findings of the study and the messages that had been identified in collaboration with the MUMS@RISC panel. The panel also had suggested some initial ideas for visuals that could accompany these messages*,* which I discussed with the illustrator. These included a pregnant woman dragging along a ball and chain to represent past trauma. At the end of our meeting*,* we agreed on a brief for each of the messages. For the message “The past does not stay in the past”*,* we needed an image to reflect that women carry their past with them*,* and their previous trauma impacts current vulnerability*,* in particular during healthcare contacts in pregnancy and early motherhood.*

**Artist**

**Fig. 1:**
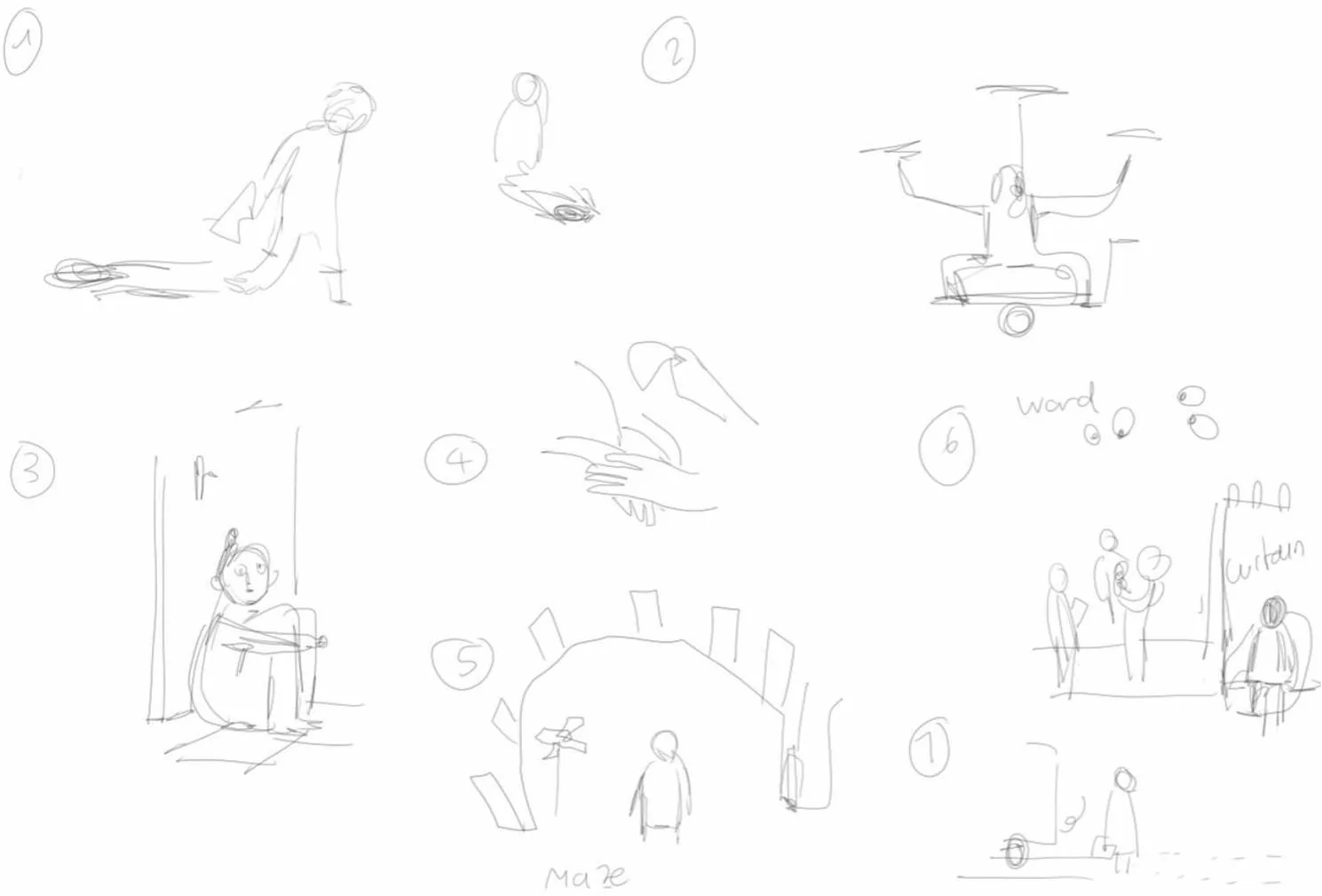
Initial digital rough sketches for illustrations from brainstorm session. **Image description**: Seven rough and unfinished drawings, most in just a few pencil lines, showing the following: (1) a person crouching forward and dragging something heavy behind them; (2) a person balancing on a plate, positioned on a ball, spinning three plates, one in each hand and then one on top of their head; (3) a person sitting on the floor, leaning against a closed door; (4) two hands holding each other; (5) a person standing in the middle of a circle, bordered by different doors, with a series of arrows pointing in different directions; (6) a person sitting behind a curtain, with other people standing on the other side of the curtain, overlooked by a series of eyes; (7) a person holding a suitcase, and a car or bus leaving them behind

*Following the initial explorative meeting with the lead researcher*,* I started sketching the “ball and chain” and other first ideas. The researcher and I had another meeting in which I showed my sketches*,* as I wanted to ensure there were not going to be any triggering elements before our creative co-design meeting with the panel.* Figure 1 * features an initial sketch for all seven messages. I also prepared a more detailed version of each of these sketches in preparation for the first meeting with the advisory panel.*

### Creative session 1

A first creative session was organised to present and discuss the first sketches, prepared by the artist. To capture the message about the ongoing impact of childhood adversity on healthcare interactions, version 1 was presented to the panel. Sketches for each of the messages were explored step by step with the panel, and feedback and suggestions were welcomed. For some of them, women provided ideas for an entirely new visualisation of the message.

**Fig. 2:**
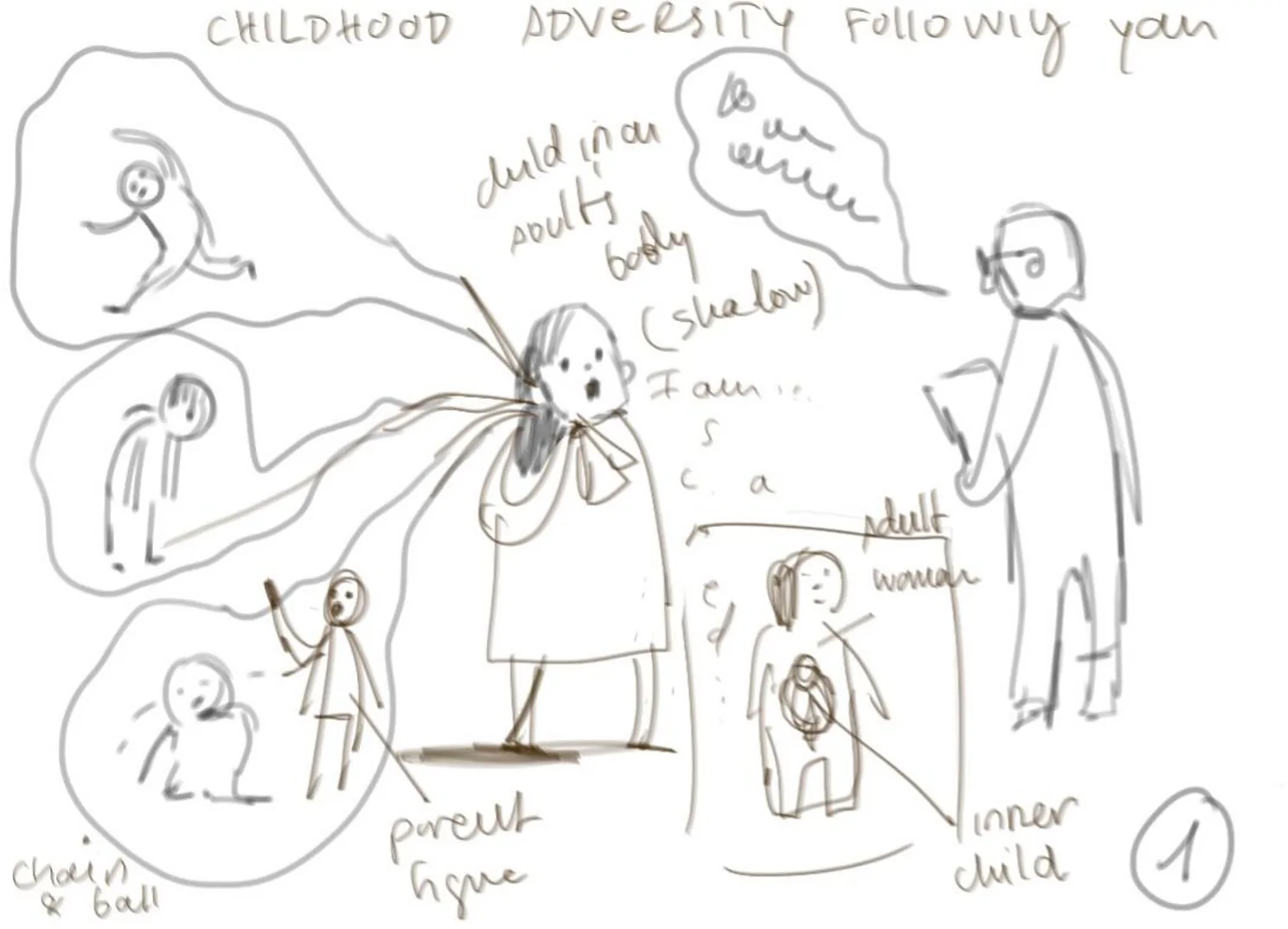
Version 1 of image representing the impact of childhood adversity on women’s healthcare interactions. **Image description**: A female person, holding on to three different balloon-type bags, each filled with a younger version of themselves showing sadness, being shouted at or running away. The female is facing a male person wearing glasses, holding paperwork, and saying something indistinct to the woman. At the bottom of the image is a smaller drawing of the woman, with her ‘inner child’ drawn inside her abdomen

**Researcher**

*During this first session, I viewed myself as a facilitator between the advisory panel members and the artist, who had not met each other before. My role during this session was two-fold: On the one hand I wanted to make sure women felt comfortable to voice their feedback and ideas; on the other I had to monitor that any ideas that were put forward were still in line with research findings and the scientific accuracy of the images was maintained. This meant at certain points I had to tone down or recalibrate any suggestions, in a respectful and constructive way, without dismissing women’s creative suggestions and ideas.*

**Artist**

*During the session, I shared my screen with the digital roughs (Fig.* *2* *) and annotated any suggestions in real time (visible in a lighter line in the images). This interactive approach allowed us to regard the drafts as a work in progress and not get fixated on certain elements too early. It was useful for everyone to see how things could work visually and how some ideas might work together. I wanted to hear if the panel members had any suggestions for specific adverse childhood events to go in the thought threads. I had attempted to present these as tentacles, but it quickly became apparent that I needed to find ways to make them look as they were weighing the woman down. I took notes about including the ‘ball and chain’ and ‘a child in an adult body image’.*

At the end of the first co-production session, the illustrator incorporated the feedback into the images, which then formed the starting point for the follow-up creative session.

### Phase 3 – Reworking the images based on feedback from creative session 1

**Artist**

**Fig. 3:**
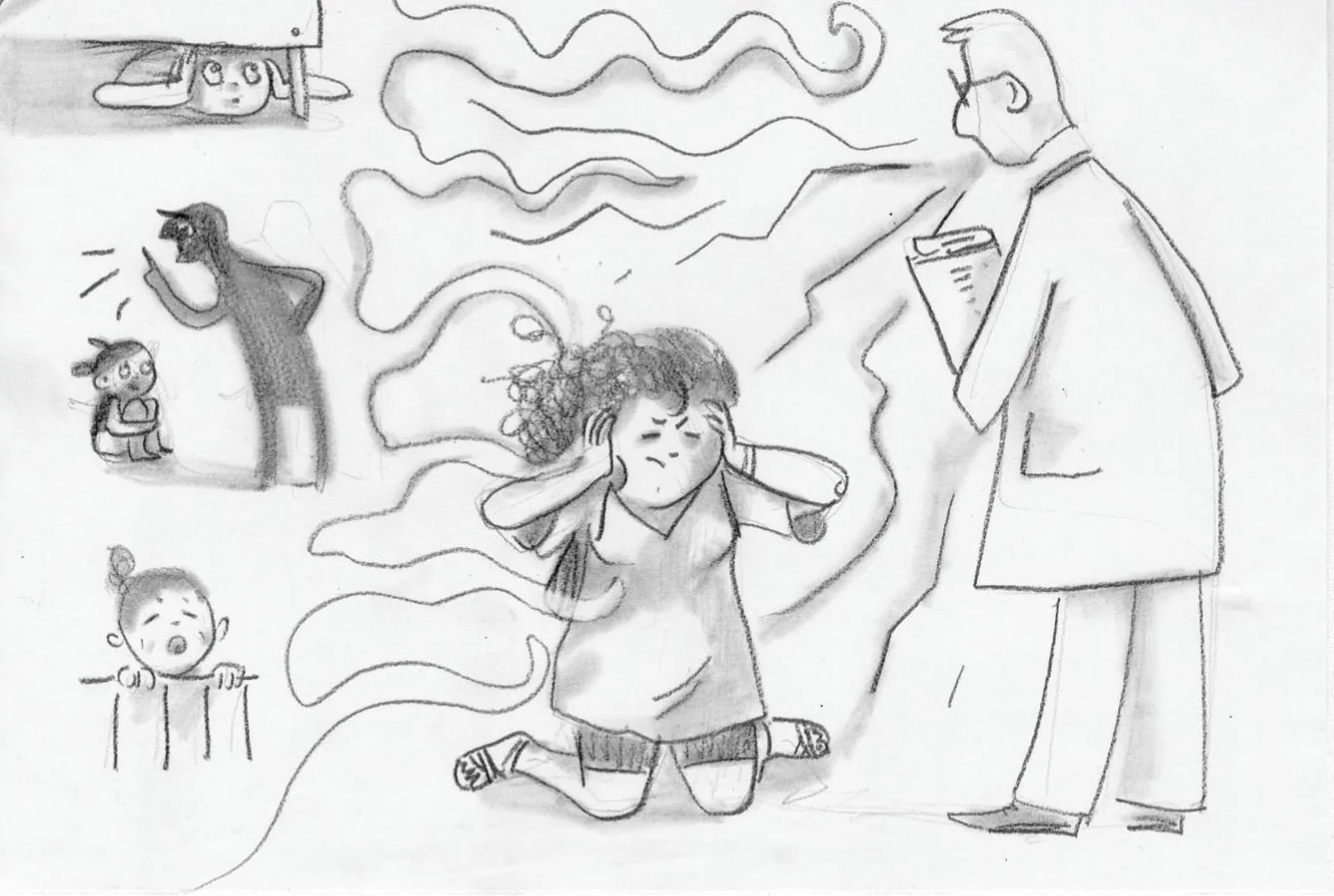
Version 2 of image. This version was discarded in view of too many (confusing) references to childhood adversity. **Image description**: A pregnant woman, wearing a dress, shorts and sandals, is kneeling on the floor, while covering her ears and looking upset. To her left are three smaller drawings of a child, one hidden under a bed, one being shouted at by a male adult, and one of the child crying standing upright her cot. Tentacle-like curves connect the images and reach out to the woman in the middle. To her right is an image of a male professional, wearing a lab-coat and holding on to documents. Waves are leaving his mouth and reach the woman

**Fig. 4:**
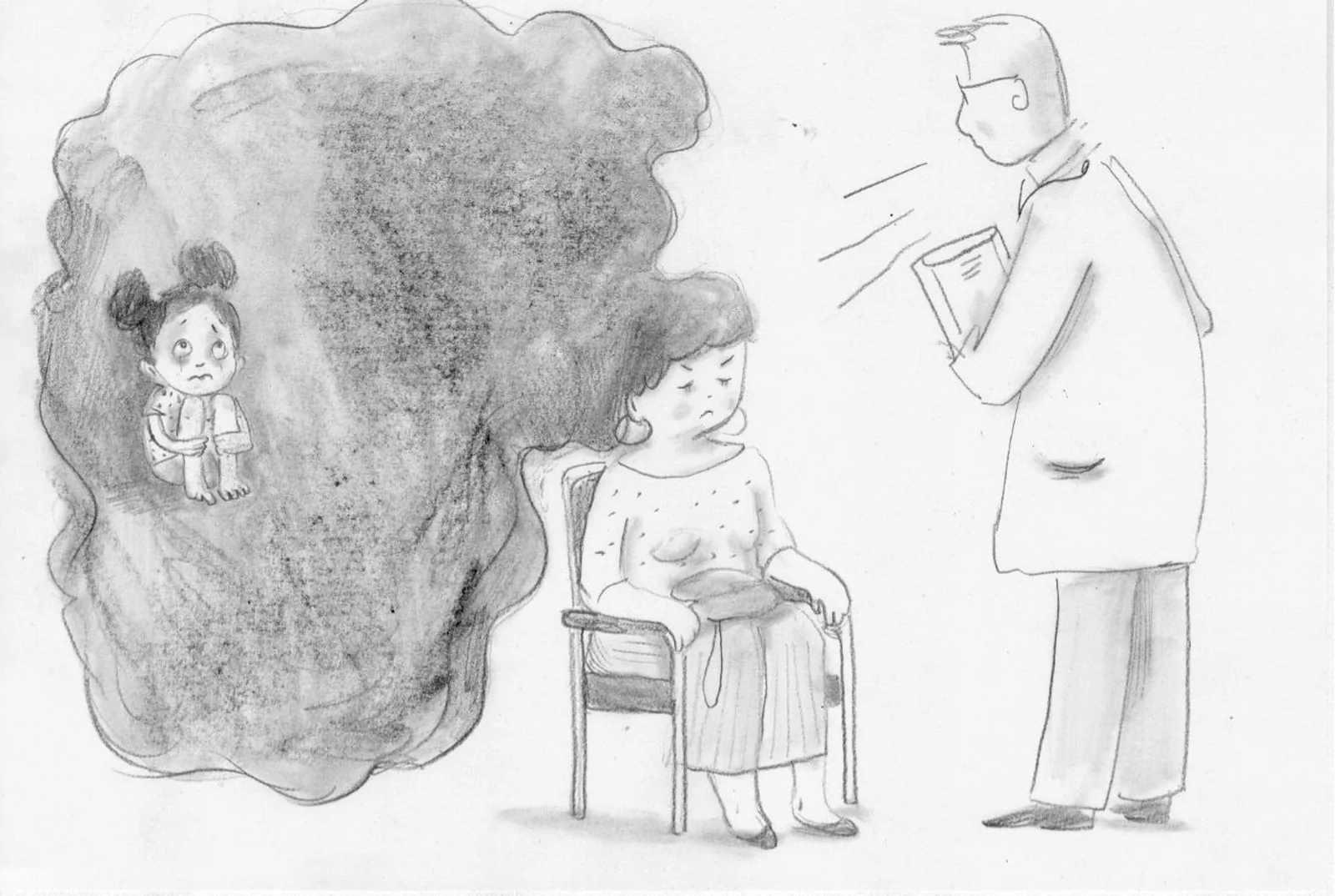
Version 3 of image, containing a single reference to childhood adversity. **Image description:** Central is a woman sitting on a chair, holding the armrests of the chair, while a handbag is resting in her lap. She is dressed in a white polkadot long-sleeved shirt, a striped ankle-length skirt, ballerina-style shoes, and large hooped earrings. The woman has closed her eyes and appears to be angry or sad. Her black hair swerves out to the left and morphs into a dark cloud. Inside the cloud, is the image of a little girl, sitting in a foetal position on the floor, looking scared and tearful, with her eyes wide open. The girl has two black ponytails and is wearing a similar polkadot shirt. To the woman’s right is a male professional, wearing a white lab-coat, glasses and holding documents. Through lines from his mouth, he appears to be speaking to her

*After the meeting,* *I created the first pencil drafts refining and cleaning up the draft sketches and incorporating some of the suggestions as seen in version 2 (Fig.* *3* *). The researcher thought version 2 was quite difficult to follow and contained too many different elements. We agreed I should explore further possibilities and incorporate some of the feedback from the session, for example by making the memories darker. I then produced version 3 (Fig.* *4* *), distilling the various representations of childhood adversity in a single image while retaining the power imbalance and clinical context.*

### Phase 4 – Creative session 2

During this follow-up creative session, the illustrator showed the reworked images and explained how she had incorporated women’s feedback. To test out the colour palette of the final images, one of the images was presented in colour, to obtain feedback on this aspect of the artwork. Women were again invited to comment and make further suggestions to improve, enhance or tweak the images, or to provide ideas for further detail or accuracy. This iterative cycle of feedback or comments provided further detail and layers of meaning.

**Panel member A**

*When we were shown the reworked images, it was clear that the illustrator had listened well to the panel and taken on board our feedback and suggestions. This was empowering, to see that our viewpoints were important enough to be reflected in this way. I could clearly see the value of having a visual representation of our experiences alongside the written research. This would prompt a visceral reaction, a feeling sense of what we went through. I felt sad when seeing the images, being reminded of the inequality I experienced engaging with authority figures during my ‘care proceedings’ process; the barrage of inexplicable information that made me feel inadequate and overwhelmed; the deep desire for it all to stop and the escalating panic that ensued. I certainly related to the woman covering her ears.*

**Panel Member B**

*When I first saw the images, I was blown away by the power that had been displayed in them. The images really reminded me of my own experience during my involvement with children social services. Seeing how the artist was able to capture the raw emotion and conviction that we spoke about was so empowering as a co-producer but also as a mother who is still healing from my interactions. This particular image is a true representation of what happens for women like myself when dealing with healthcare professionals in these situations. We feel intimidated and go back to our inner child.*

During the discussion, panel member gave further feedback on the finer detail of the image. They loved the position of the woman in a clinical chair, with the healthcare professional in a superior position, towering over her, and preferred this to the woman’s previous ‘crouching’ position. They also made further suggestions to the artist to ensure the little girl looked like the woman in the chair and truly resembled women like themselves:

- Panel members suggested that the woman appeared too middle class, by means of her clothes, her shoes and her handbag. Her current appearance did not resonate with panel members;
- Panel members wanted to see more similarities between the woman and her younger self, for instance through hair, eyes, or other facial features. They also suggested this might be achieved by placing her in a similar position as the little girl, for example by holding her abdomen in the same way the girl was holding her knees.

**Researcher**

*During this second session, it was exciting to see that the women were impressed by how the illustrator had incorporated their feedback. During the brief period of collaboration between the panel and the illustrator, it was clear to me they trusted her and felt heard and listened to. It also meant that during this second session, women were eager to bring further suggestions and additions to the images, based on their own lived experience. There was a risk that the numerous suggestions from various panel members would detract or dilute the message, or the final image might digress from the actual research findings. As such, my role during this follow-up session was less of a facilitator to establish trust, but more as a mediator, to ensure only those suggestions that were in line with the research findings would be further explored.*

**Artist**

*I was again surprised by the sophisticated and thoughtful feedback the panel provided. I took steps to make the cloud darker and worked on the resemblance in the posture*,* eyes*,* mouths and the parting of the hair. I took on board the recommendation to make the clothing less formal or middle class. The panel had suggested leggings*,* jeans and simple tops as items typically worn instead of elegant skirts and feminine accessories. During the meeting*,* I made notes in the form of small sketches*,* one of a woman holding her pregnant belly and the girl hugged her legs and looked for ways to incorporate these elements in the image*,* removing the handbag in the process and other unnecessary details that could distract from the main message.*

### Phase 5 – Final comments and approval

**Fig. 5:**
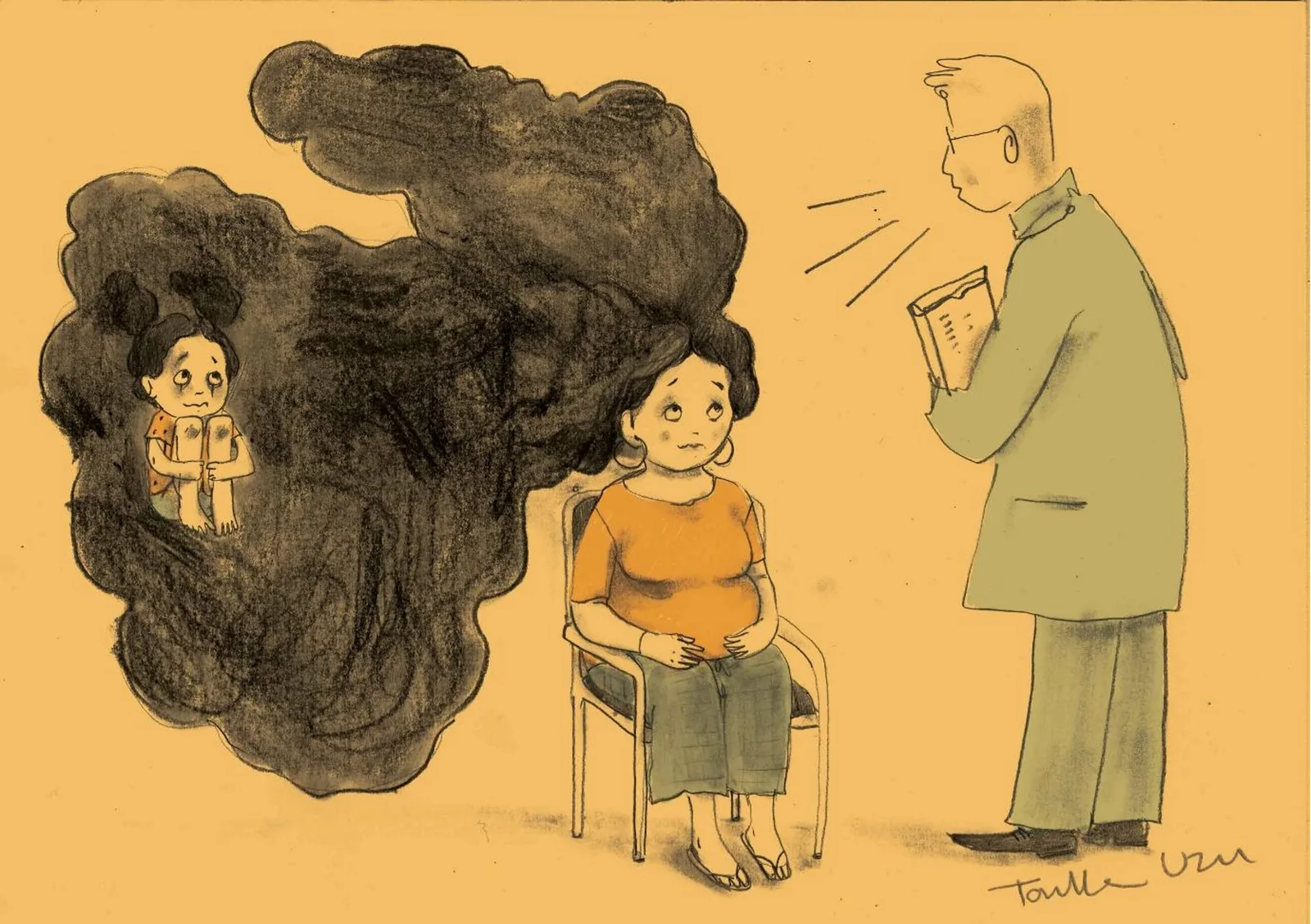
Final illustration in colour. **Image description:** Central is a woman sitting on a chair, with her arms wrapped around her pregnant belly. She is dressed in blue trousers, a plain orange t-shirt, flipflops, and large hooped earrings. The woman is looking wide-eyed at the male professional in front of her and her mouth curved into an anxious, tight shape, almost as if she is biting her lip. Her black hair swerves out to the left and morphs into a dark cloud, shaped in two parts, almost like two ponytails. Inside the cloud, is the image of a little girl, sitting in a foetal position on the floor. The girl is similarly dressed, in an orange polkadot t-shirt and blue shorts. Her knees are dirty and she is looking scared and tearful, with her eyes and mouth almost shaped identical as the grown woman in the chair. The girl has two black ponytails, reflecting a similar shape as the woman’s hair. To the woman’s right is a male professional, wearing a light green lab-coat, green trousers, glasses and holding documents. Through lines from his mouth, he appears to be speaking to her

The images were shared via email with all the women, and a separate session was organised without the illustrator present to discuss them for any final tweaks or changes. This stage of the images is reflected in version 4 (Fig. 5). It was a deliberate decision to hold this session without the illustrator, as both the researcher and illustrator were aware that some women might refrain from voicing their feedback in the illustrator’s presence, out of fear of upsetting her, or being disrespectful. It was important to ensure that all panel members felt comfortable to freely express their views on the re-worked images.

**Panel Member A**

*As the illustrations developed, the ‘mother’ gained a sense of composure, appearing to mask the inner turmoil whilst her inner child shook with fear. I remember the panel sharing the incongruence felt at having to ‘pretend’ everything was alright when interacting with professionals, the pain this caused and the utter exhaustion at being surveilled throughout pregnancy.*

**Panel Member B**

*I was blown away by the process, to me this truly has been one of the most restorative and healing parts of this research project. Seeing how an image can truly say a thousand words, the raw emotion that the image invoked and being able to be a part of a special group of women including the researcher, has been so powerful. This is where the true strength of the image lies: Although none of the images look like me, I can see myself in them and all the other women that I work with or support.*

After the session, the researcher shared any final feedback with the Illustrator and comments were particularly positive about the following aspects of the final image:

⦁ How both the adult and child version of the women resembled one another, through subtle but important details, such as hair texture, the look in their eyes, and the shape of their mouth.

⦁ The changes to the clothing, by removing the handbag and introducing jeans and flip-flops.

⦁ The colour scheme, warm but also monochrome in a way.

All images were unanimously approved by all the members of the advisory panel.

**Artist**

*Having a final design approved is a welcome point in any commission. On this occasion there was an added sense of anticipation*,* given that I was keen to know whether the panel felt that their ideas and suggestions had been successfully translated in the images and that they felt they were a truthful representation of their lived experience. The aspect of the clothing was particularly interesting to me*,* as I had deliberately depicted the women in nice clothing*,* as I felt the need to avoid stereotypes. Yet*,* the women in the panel felt that this depiction was not authentic and showed how pointless it is to assume what would be regarded as offensive without consulting those with lived experience. The collaboration felt productive and inspiring throughout*,* thanks to the careful planning of the researcher. It was also invaluable in the last stages that the researcher was available to explain any creative decisions*,* for example regarding elements that had to be left behind for clarity and composition purposes. Reflecting back*,* I have been surprised by the simplicity we achieved in in the final image*,* which went in the opposite direction of my initial sketches*,* which focused on distinct scenes of traumatic childhood events. Through the cyclical involvement from the panel*,* we managed to create an image that is powerful in its simplicity to depict the inner child/past self of the woman and its impact on her pregnancy care.*

### Dissemination of the artwork

After final approvals, the artwork was incorporated in the summary report of the study [18] and has also used in conference presentations for difference audiences from a range of backgrounds, including academia, health and social care, third sector organisation, lived experience representatives as well as policy makers. Informal audience feedback has been unanimously positive, praising the illustrations for their emotive and powerful appeal.

**Panel Member B**

*When we presented the images at a co-produced event, the reaction from professionals was exactly what we wanted to convey, to remind them that women carry a lot more than what is shown on the surface. This process from start to finish made me feel heard and seen, valued and thought about and as such, has been a big part of my healing and rewriting my story.*

### Reflections and recommendations

The MUMS@RISC advisory panel were instrumental to the MUMS@RISC study and its wider impact. Their continued involvement throughout the entire study demonstrated commitment, advocacy, and an insightful understanding of the challenges around study recruitment, analysis, and dissemination, while simultaneously challenging persisting narratives about women who experienced custody loss as ‘too hard to engage’ or difficult to retain in a research advisory panel. Our reflections capture the key elements of successful trauma-informed research collaboration and participation, with recommendations for future research in this area.

- Early establishment of trusting relationships with relevant third sector organisations and experts by experience is key to genuine and meaningful research collaboration. Building these relationships takes time and should ideally be initiated before the start of a study, but this can be hindered due to financial and logistical restrictions in the pre-award stage.
- Meaningful research collaboration starts with allocating sufficient time to foster a safe and empowering space for the advisory panel to come together. The creation of the MUMS@RISC charter for research engagement took multiple advisory meetings, but was a deliberate choice to not rush into discussions about study design or recruitment materials, as may be often the case during the early stages of a research project. It fostered trust that was essential for continued involvement of the panel members, when they so often had been let down by professionals, or had felt rushed in administrative processes. It also offered an opportunity to align ourselves as one study team, with core values that we all aimed to uphold. This may have contributed to a shared sense of ownership of the study and trust in the research team.
- Trauma-informed research collaboration and participation is led by those with lived experience, as they are best placed to understand the pitfalls and danger points in a study design for potential re-traumatisation. The diverse composition of the advisory panel also meant that different viewpoints were continuously heard, making for a robust understanding of how the emotional wellbeing of research participants as well as panel members could be safeguarded as much as possible throughout research participation and engagement. The continued support from relevant third sector organisation was also critical in this respect, to ensure post-interview support remained available to research participants if this was required. Future research studies in this field may design a different safety protocol, but as long as such a protocol is co-designed with experts by experience and wider involvement from key stakeholders that can provide support and assistance, it will be invaluable to ensure the safety and wellbeing of potential research participants.
- The MUMS@RISC advisory member played an invaluable role in the dissemination strategy of the study findings and the co-produced artwork is testament to their involvement, passion and creativity, as well as to the artist’s ability to listen to feedback and translate that into artwork. The creative process was for all involved a respectful, safe and empowering collaboration, resulting in high quality and powerful artwork, that has struck a chord with both policy, clinical and lay audiences. It has highlighted the importance of preparing and incorporating this aspect of the research cycle with care and creativity, and not consider it as frivolous afterthought when disseminating research findings.

## Conclusion

Trauma-informed and robust lived experience involvement in studies about sensitive issues is not only a prerequisite for rigorous, in-depth and meaningful research. Our approach to research collaboration and participation has shown that by centring the voices of women, who have so often been dismissed or marginalised, and by applying creative methods for disseminating research findings, research contributors can feel empowered, and for some, this even has been a healing experience. The cyclical involvement of lived experience voices has ensured that complex mixed methods research findings were distilled into powerful artwork, while also being rooted in women’s own experiences. These powerful images will stay with readers and viewers, long after statistical results or qualitative themes have been forgotten.

## Supplementary Information

Below is the link to the electronic supplementary material.

Supplementary Material 1

## Data Availability

No datasets were generated or analysed during the current study.
