# Supplementary material for: Centring the voices of birth mothers who faced infant removal through the co-production of the MUMS@RISC artwork: an arts-based evaluation of trauma-informed research collaboration and participation
Source: Res Involv Engagem. 2026 Sep 17;12:148. doi: 10.1186/s40900-026-00966-6 (PMC13584445; doi:10.1186/s40900-026-00966-6)
Supplement: Supplementary file 1 [file 40900_2026_966_MOESM1_ESM.pdf]

SUPPLEMENTARY MATERIAL S1 – INFORMATION LEAFLET FOR RECRUITMENT OF ADVISORY PANEL MEMBERS

MUMS@RISC study

IMPROVING CARE FOR WOMEN FACING REMOVAL AT BIRTH

FUNDED BY NIHR | National Institute for Health and Care Research

Hi there,

Please let me introduce myself.

My name is [redacted] and I am a midwife by background and started a PhD in October 2022. As a midwife, I cared for a caseload of women with mental health difficulties and some of my caseload women had their baby removed due to care proceedings shortly after birth. I wanted to do something to improve care for mothers who go through care proceedings and hope to achieve this through my research.

For my research, I am keen to speak to women who have been separated from their baby due to social care proceedings, to understand their care experiences, needs and expectations and how we can make care better. Perhaps this is something you might be interested in?

What will happen if you take part?

I would like to have two conversations with you. First, to hear about anything that happened to you before you got pregnant.

Second, to hear about the support and care you had during pregnancy and the postnatal period.

Participants will be reimbursed for their time and any travel or childcare expenses.

Please consider participating if you are …

- 18years or older
- You had healthcare during pregnancy)like midwifery care,…)
- You were separated from your baby in the first month after you gave birth due to social care proceedings, including via section 20 arrangements
- This happened less than 5 years ago
- You feel ready to share your story

If you are interested in becoming a member of the expert panel, don’t hesitate to get in touch.

MUMS@RISC study

IMPROVING CARE FOR WOMEN FACING REMOVAL AT BIRTH

Charter for research engagement

This charter brings together the values that underpin the MUMS@RISC study and we strive to reflect these values in all parts of the research, such as the research design, our interaction with research collaborators and any research communication. The charter was developed by the MUMS@RISC advisory panel, consisting of mothers who experienced removal of their baby after birth and inspired by the Charter for survivor engagement of Survivors' Voices.

- **Respectful, safe and Caring**: A respectful, safe and caring environment is crucial to any research engagement. Everyone is unique with their own story to tell, and we welcome, respect, and celebrate people's diverse backgrounds, experiences, knowledge and skills.

- **Being heard, seen and valued**: We acknowledge that many collaborators will have been on the receiving end of dehumanising processes. Their problems became the only thing that seemed to matter to others and they were no longer seen as a person with a voice and feelings. Our research engagement wants to be person-centered, not problem-centered.

- **Empowering**: We acknowledge that women too often have been silenced and disempowered, with little control of their own narrative. Experts by experience must be empowered to have control over decisions about their own involvement. Research engagement should be positive and give women a voice to express what change they wish to see and explore how they feel this can be achieved.

- **Accountable, transparent and effective**: There is no 'hidden' agenda with this research: all communication should be transparent and clear. Processes and decision-making should be relational, honest, real, transparent and open to feedback and dialogue.

Supplementary material S2 – Charter for Research Engagement

- **Trauma-informed**: We acknowledge that experiences of trauma have shaped the lives of many of research collaborators and participants and that participating in research engagement might trigger some of these traumatic experiences. We strive for a research environment where one feels safe and we aim to avoid such triggers, intentionally and unintentionally. Where required, women can access additional support and safety protocols are in place to ensure the psychological safety of all involved.

- **Poverty-proof:** We acknowledge that the cost-of-living crisis is disproportionately affecting households on lower incomes and that this creates additional obstacles in participating in public life, and by extension research engagement. Any effort will be made to enable research collaboration and avoid 'un-noticed' out of pocket expenses, such as joining meetings online on their phone using their own data, travel costs, etc. Collaborators are able to decide how they would like to be reimbursed for their time based on their own financial circumstances (i.e. transfer, vouchers, combination) and can access advice from a specially trained Citizen Advise Bureau Advisor with expertise in this area.

- **Accessible and inclusive:** Research should be accessible and inclusive to all, and so should the outputs of research. We strive to create accessible, inclusive and appealing research outputs, to amplify the voices of those that participated, while avoiding jargon.

- **Hopeful:** We acknowledge that research collaborators often have lived very difficult lives, marked by trauma, and exacerbated by the grief of the loss of their child(ren). Meaningful research engagement should aim to instil hope for change and a better future and celebrate the contributions of those involved.

- **On this journey together**: None of us are perfect and we jointly learn how to make this process more worthwhile and enduring. There is a joint commitment to continue to listen and learn from one another and to ensure the values in this charter are upheld throughout the entire research journey.

Supplementary material S3 – Trauma-informed Research Collaboration and Participation

The following list provides an (non-exhaustive) list of how trauma-informed principles were adopted throughout the MUMS@RISC study:

1) **Realising** the prevalence of trauma
- Awareness of previous trauma among panel members and research participants
- Awareness that previous traumas might still be unprocessed, or actively ongoing
- Awareness that conducting this research might also be triggering for the researcher involved

2) **Recognising** how trauma affects individuals
- Awareness that panel members or research participants might display self-protective behaviours when feeling threatened, vulnerable or not respond to email or phone calls
- Awareness that panel members might have different views about sharing their own experiences, with some having the need to share detailed accounts, and others being apprehensive to talk about their experiences
- Debriefs offered to panel members and research participants
- Ensuring third sector organisations were available to provide continued and enhanced support if needed

3) Actively **resisting** re-traumatisation through carefully considered processes and settings
- Careful consideration of language used in recruitment materials, during panel meetings, or during interviews with research participants
- Providing as much control and feedback mechanisms to panel members and research participants, by returning meeting notes (panel meetings) or transcripts, core stories and draft manuscripts (research participants).
- Providing iterative cycles of sense-checking to give participants repeated opportunity to feel they are in control and their voice matters
- Trauma-informed interviewing training completed by the lead researcher prior to the start of the study
- Clinical experience of the lead researcher in trauma-informed care (through their clinical experience as a specialist mental health midwife) and having meaningful conversations with women on sensitive topics
- Composition of a safety protocol, with clear escalation processes in case of distress and concerns about mental wellbeing of research participants or panel members
- Check-ins at start and end of panel meetings
- Debriefs with research participants within 48hrs after interview

4) **Responding** by putting this knowledge into practice
- Upholding the group agreement and Charter for Research Engagement at all times
- Continuous reflections on how to ensure panel members and research participants were safe, valued and empowered.

SUPPLEMENTARY MATERIAL S4 – ITERATIONS OF ILLUSTRATIONS

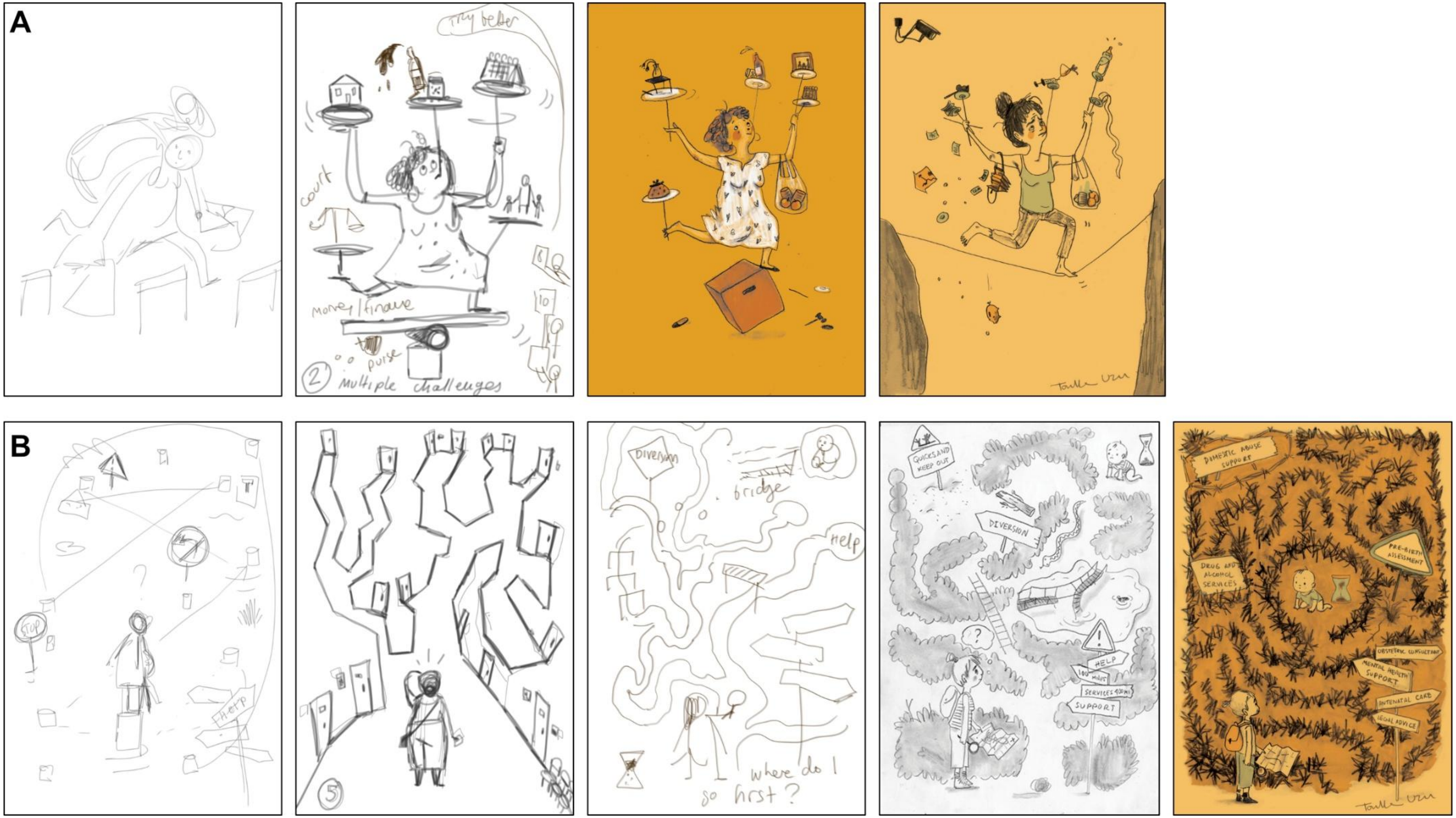

Supplementary material S4: Iterations of two further illustrations of the MUMS@RISC artwork. A. Key message: Current care pathways are not fit for purpose for women with complex adversity. **B.** Key message: Multi-agency systems are challenging and complex.
